# Participants’ Engagement With and Results From a Web-Based Integrative Population Mental Wellness Program (CHAMindWell) During the COVID-19 Pandemic: Program Evaluation Study

**DOI:** 10.2196/48112

**Published:** 2023-10-26

**Authors:** Joseph A Rosansky, Kayley Okst, Miriam C Tepper, Ana Baumgart Schreck, Carl Fulwiler, Philip S Wang, Zev Schuman-Olivier

**Affiliations:** 1 Department of Psychiatry Cambridge Health Alliance Cambridge, MA United States; 2 Department of Psychiatry Harvard Medical School Boston, MA United States; 3 Department of Psychology New York University New York, NY United States; 4 New York State Psychiatric Institute Columbia University New York, NY United States; 5 Department of Psychiatry Brigham and Women’s Hospital Boston, MA United States

**Keywords:** COVID-19 pandemic, digital psychiatry, early identification, integrative medicine, mental wellness, mindfulness, population mental health, prevention, stratified care

## Abstract

**Background:**

The COVID-19 pandemic involved a prolonged period of collective trauma and stress during which substantial increases in mental health concerns, like depression and anxiety, were observed across the population. In this context, CHAMindWell was developed as a web-based intervention to improve resilience and reduce symptom severity among a public health care system’s patient population.

**Objective:**

This program evaluation was conducted to explore participants’ engagement with and outcomes from CHAMindWell by retrospectively examining demographic information and mental health symptom severity scores throughout program participation.

**Methods:**

We examined participants’ symptom severity scores from repeated, web-based symptom screenings through Computerized Adaptive Testing for Mental Health (CAT-MH) surveys, and categorized participants into symptom severity-based tiers (tier 1=asymptomatic to mild; tier 2=moderate; and tier 3=severe). Participants were provided tier-based mindfulness resources, treatment recommendations, and referrals. Logistic regressions were conducted to evaluate associations between demographic variables and survey completion. The McNemar exact test and paired sample *t* tests were performed to evaluate changes in the numbers of participants in tier 1 versus tier 2 or 3 and changes in depression, anxiety, and posttraumatic stress disorder severity scores between baseline and follow-up.

**Results:**

The program enrolled 903 participants (664/903, 73.5% female; 556/903, 61.6% White; 113/903, 12.5% Black; 84/903, 9.3% Asian; 7/903, 0.8% Native; 36/903, 4% other; and 227/903, 25.1% Hispanic) between December 16, 2020, and March 17, 2022. Of those, 623 (69%) completed a baseline CAT-MH survey, and 196 completed at least one follow-up survey 3 to 6 months after baseline. White racial identity was associated with completing baseline CAT-MH (odds ratio [OR] 1.80, 95% CI 1.14-2.84; *P*=.01). Participants’ odds of having symptom severity below the clinical threshold (ie, tier 1) were significantly greater at follow-up (OR 2.60, 95% CI 1.40-5.08; *P*=.001), and significant reductions were observed across symptom domains over time.

**Conclusions:**

CHAMindWell is associated with reduced severity of mental health symptoms. Future work should aim to address program engagement inequities and attrition and compare the impacts of CHAMindWell to a control condition to better characterize its effects.

## Introduction

The COVID-19 pandemic involved a prolonged period of collective trauma and stress during which mental health concerns, such as depression and anxiety, increased across the population [1,2]. During and after collective trauma, the need for acute psychological services is widespread [3], but those affected by disasters (eg, Hurricane Katrina) are often undertreated due to a lack of access to services and stigma associated with psychiatric treatment [4]. Additionally, during the last major pandemic before COVID-19, the Spanish flu pandemic of 1918, increased social distancing was associated with increased suicide rates, irrespective of actual flu mortality rates [5].

These gaps were also observed during the COVID-19 pandemic, with substantial increases in depression and anxiety and a simultaneous reduction in the use of outpatient mental health treatment at the start of the pandemic [2,6]. Furthermore, a meta-analysis of 54 investigations found that rates of suicidal ideation, suicide attempts, and self-harm increased during the pandemic compared to the year before the pandemic began [7]. A variety of factors may have contributed to this, including reduced access to traditional behavioral health treatments due to pandemic precautions and provider burnout, challenges implementing and accessing remote care, and ongoing stigma related to using behavioral health care [8,9]. In this context, there was an urgent need to develop alternative approaches to providing population behavioral health support.

Previous research has identified stratified mental health care as an approach that can maximize care system resources while effectively treating patients’ symptoms [10,11]. This approach uses decision tools to assign patients to the interventions best suited to address their specific symptoms. Stepped-care approaches initially assign all patients to similar low-intensity treatments and then increase treatment intensity for those who do not respond, which also appears to reduce costs and promote recovery and has been more commonly used than stratified care [12]. However, stepped-care approaches appear to be less effective for patients with more complex presentations (eg, greater symptom severity, disability, younger age, and unemployment), and a recent cluster-randomized trial of 951 patients in the United Kingdom found that stratified care was associated with improved outcomes [10,13]. These findings could reflect that stratified care approaches avoid delays in getting individuals to their appropriate levels of treatment and do not require patients to fail lower levels of care first.

While stratified care models can help lower costs, the expenses associated with providing behavioral health support to large groups of people can be prohibitive unless cost-effective treatment approaches are used. Providing individuals with educational materials about ways to improve their mental health (ie, passive psychoeducation) is one low-cost, evidence-based approach that has been shown to reduce symptoms of depression [14]. Self-guided behavioral health treatment apps that use cognitive behavioral- and mindfulness-based treatment approaches are another low-cost approach that has been shown to promote symptom reduction [15,16]. In addition, regular remote symptom assessments and personalized feedback after stratification-based treatment assignments appear to be promising tools for improving patient outcomes in mental health care settings, and many aspects of these types of interventions can be automated and incorporated into a measurement-based care approach [17-19]. We, therefore, attempted to incorporate these elements into CHAMindWell, an integrative population mental wellness program using a semiautomated approach incorporating elements of stratified and measurement-based care that was developed in response to the COVID-19 pandemic at a large public safety net health care system in Massachusetts serving a catchment area of 450,000 people. In this program evaluation, we sought to assess participants’ engagement with the CHAMindWell program as well as the impacts of program participation on participants’ mental health symptoms.

## Methods

### Program Design and Implementation

#### Overview

We designed CHAMindWell to prevent the onset or worsening of mental health problems among healthy participants and facilitate early identification and treatment to reduce symptom severity among those with clinical concerns. To access mental health care resources before this program’s development, patients needed to be referred to the hospital system’s outpatient psychiatry department or primary care behavioral health integration and complete a psychiatric diagnostic assessment in a stepped-care model. To reduce the burden that a population mental wellness program focused on early identification might place on traditional mental health services (eg, individual therapy) while still meeting the treatment needs of participants with more complex presentations, CHAMindWell used a stratified care model. The program included components of symptom monitoring, motivational interviewing, psychoeducation, coaching, app recommendations, safety assessments, referrals to mindfulness training and mindfulness-based group therapies, and referrals to other internal and external treatment sources. We made several mindfulness-based group services and resources available to CHAMindWell participants because these interventions have been shown to effectively reduce stress, anxiety, and depression and increase resilience while being acceptable for web-based delivery [20-22].

Within 1 week after the first COVID-19 lockdown in Massachusetts, we initiated preparations for a pandemic-related mental health surge. We targeted this program toward the hospital system’s primary care population and patients who had been placed on waitlists for behavioral health services. To recruit these individuals, we periodically sent them emails and messages through the electronic health record’s patient portal (MyChart) with information about CHAMindWell. We also educated primary care providers about the program and developed a website where potential participants could learn about and register for CHAMindWell [23].

Participants in CHAMindWell completed recurring surveys assessing mental health symptom severity using Computerized Adaptive Testing for Mental Health (CAT-MH) modules [24]. The modules assessed for symptoms of depression, anxiety, posttraumatic stress disorder (PTSD), psychosis, mania or hypomania, and the risk of developing a substance use disorder. CAT-MH used multidimensional item response theory procedures to adaptively select questions from each module’s item bank based on a participant’s previous responses to efficiently obtain a precise estimate of symptom severity, quantified by a severity score between 0 and 100 [25]. Although a module assessing suicide risk was initially included, we discontinued that module because it was designed to predict the risk of developing suicidality over the next 6 months rather than describe immediate risk [26,27].

To ensure confidentiality, meet hospital information security requirements, and increase efficiency in screening large numbers of individuals, we developed an open-access Research Electronic Data Capture (REDCap) external module to distribute CAT-MH surveys and paired it with a coordinator dashboard to manage daily monitoring of results [28,29]. The coordinator dashboard included data such as completion time, missing assessments, and CAT-MH results for each participant. We also collected participant feedback, both formal (eg, Patient and Family Advisory Committee) and informal (eg, through phone contact with participants), to iteratively improve the user experience and overall program design.

Once enrolled, participants in CHAMindWell were asked to complete CAT-MH survey batteries at baseline and months 1, 2, 3, 4, 6, 9, and 12. In response to participant feedback about the frequency of survey invitations, in June 2022, we reduced the survey invitation frequency to baseline and months 2, 4, 6, 9, and 12. Project coordinators reviewed CAT-MH survey results on a rolling basis and assigned each participant to 1 of 3 tiers based on their most recent results. Symptom severity cut points for each tier varied by CAT-MH module, and participants were assigned an overall tier equal to their highest tiered domain (eg, depression or anxiety). Participants’ tiers were updated each time they completed a CAT-MH survey, and they were provided individually tailored, tier-based services.

#### Tier 1 Interventions

Participants with low-level symptom severity scores that did not meet clinical thresholds (eg, severity score <50; specific cut points varied by module) were assigned to tier 1. These participants were offered phone support from a coordinator trained in motivational interviewing, including guidance on using a clinician-reviewed list of self-help apps and websites, information about free access to 8-week mindfulness courses (5-10 free spots available in each of 12 groups per year), and community-based mindfulness programs offered through the hospital’s Center for Mindfulness and Compassion. Participants were also sent monthly email newsletters that included information about CHAMindWell services and psychoeducation on rotating mental wellness topics, such as seasonal changes in affect, impacts of diet on mental health, behavioral changes to improve sleep, and related subjects.

#### Tier 2 Interventions

Participants with moderate to moderately severe symptoms (eg, severity score between 50 and 75) were assigned to tier 2. They received all interventions offered to those in tier 1 and were also offered a 20-minute web-based mental wellness check-in with a clinician. These mental wellness check-ins were used to confirm symptoms indicated by CAT-MH scores (eg, anxiety, depression, psychosis, and mania) and to further evaluate participants’ treatment needs in the context of their unique circumstances. Clinicians evaluated for safety concerns, reviewed participants’ most recent CAT-MH survey results, discussed current symptoms, and provided brief coaching and app recommendations. Based on participants’ needs and interests, clinicians were able to provide referrals to relevant clinical trials and group-based mental health services. Clinicians were encouraged to refer these participants to group-based services unless symptoms indicated a clear need for higher levels of care or they voiced strong preferences for individual treatment.

#### Tier 3 Interventions

Participants endorsing severe mental health symptoms (eg, severity score ≥75) were assigned to tier 3. These participants were provided all the interventions offered to those in tiers 1 and 2, but clinicians were encouraged to also offer tier 3 participants referrals to traditional behavioral health services (eg, individual therapy and psychopharmacological treatment). Participants in need of urgent or specialty care (eg, suicidal ideation, psychosis, severe substance use, or severe mania) were referred to appropriate hospital-based treatment programs.

This program evaluation assessed participants’ engagement with and outcomes from the CHAMindWell program. We evaluated (1) whether demographic variables were associated with different levels of program engagement, (2) whether participants’ symptoms improved over the course of program participation, and (3) which types of referrals were placed for participants with clinically relevant mental health symptoms.

### Study Design

We retrospectively analyzed demographic and mental health symptom data collected from program participants who enrolled between December 16, 2020, and March 17, 2022, through the CHAMindWell consent and registration form and completed CAT-MH surveys. To preserve the methodological integrity of relevant clinical trials, participants who elected to join a trial were removed from CHAMindWell until their research participation concluded.

### Participants

Individuals were eligible to enroll in CHAMindWell if they were 18 years or older, a primary care patient or staff member at Cambridge Health Alliance, and spoke English fluently. English fluency was required for enrollment because participants needed to be able to comprehend and complete web-based surveys in English.

To examine the effects of CHAMindWell on mental health symptoms, we analyzed data from participants who completed both a baseline and a follow-up survey between 3 and 6 months later. Because some participants did not complete certain scheduled CAT-MH surveys and others did not open and complete surveys until weeks or months after receiving invitations, we used responses completed at least 75 days after baseline and as close to 180 days after baseline as possible for each participant’s follow-up assessment.

### Measures

#### Demographic Information

Participants were asked to provide their ages, gender identities, and racial and ethnic backgrounds. Participants were able to select more than one racial and ethnic background from a provided list (Table 1).

**Table 1:** Participant characteristics by program engagement status. Participants could select more than one racial and ethnic background. Besides the association noted in the table, logistic regressions did not reveal any significant associations between demographic characteristics and follow-up status.

Variable		Enrolled (n=903)	Completed baseline (n=623)	Remained enrolled after baseline (n=536)	Completed 3-6-month follow-up (n=196)
**Gender, n (%)**					
	Female	664 (73.5)	460 (73.8)	393 (73.3)	144 (73.5)
	Male	196 (21.7)	131 (21)	120 (22.4)	43 (21.9)
	Genderqueer	20 (2.2)	17 (2.7)	11 (2.1)	3 (1.5)
	Prefer not to say	14 (1.6)	6 (9.6)	4 (0.7)	2 (1)
	Missing	9 (1)	9 (1.4)	8 (1.5)	4 (2)
**Racial and ethnic background, n (%)**					
	White^a^	556 (61.6)	413 (66.3)	350 (65.3)	139 (70.9)
	Black, African American, or Haitian	113 (12.5)	63 (10.1)	57 (10.6)	18 (9.2)
	Asian or Indian	84 (9.3)	60 (9.6)	50 (9.3)	19 (9.7)
	Native American or Alaskan Native	7 (0.8)	3 (0.5)	2 (0.4)	1 (0.5)
	Other	36 (4)	22 (3.5)	19 (3.5)	5 (2.6)
	Hispanic	227 (25.1)	152 (24.4)	128 (23.9)	41 (20.9)
	Missing	41 (4.5)	24 (3.9)	22 (4.1)	5 (2.6)
Age (years), mean (SD)		39.4 (13.0)	39.6 (12.7)	39.6 (12.8)	42.0 (13.2)

^a^The variable was significantly associated with increased odds of completing baseline CAT-MH; *P*<.05.

#### Framework of CAT-MH

Each CAT-MH [30,31] module included a large, domain-specific item bank from which items were adaptively selected using multidimensional item response theory procedures. Symptom severity scores ranging from 0 (asymptomatic) to 100 (greatest severity) were calculated for each module. Most modules assessed symptom severity within the past 2 weeks. However, the substance use disorder module measured the risk of having or developing a substance use disorder over the next 6 months rather than current symptoms of substance use [32]. This program evaluation examined participants’ baseline and follow-up scores for the depression, anxiety, and PTSD modules because we felt these domains were most likely to be impacted by mental wellness support.

### Data Analysis

To evaluate whether participants’ demographic characteristics were associated with degrees of program engagement, we conducted 2 logistic regressions. The completion of at least one CAT-MH survey was the dependent variable for the first regression, and the completion of at least one CAT-MH survey 3-6 months after baseline was the dependent variable for the second. Both dependent variables were dummy-coded (0=not completed and 1=completed). For both regressions, age, gender, and racial and ethnic identities were included as independent variables.

Overall, 19.7% (178/903) of participants were missing data for age or gender. Missing data were due to participants not answering questions and changes made to the enrollment form as the program evolved (eg, participants who enrolled before March 16, 2021, were not asked to provide their ages). Therefore, we performed each regression twice, first using only complete cases and then imputing missing data using the *mice* (version 3.14.8; multivariate imputation by chained equations) package in R statistics (R Core Team) [33]. This package used fully conditional specification to separately impute missing values for each independent variable used in the regressions. We used predictive mean matching to impute missing values for age (173/903, 19.2% missing) and polytomous logistic regression for gender (9/903, 1% missing). We repeated this procedure to create 5 complete data sets, performed logistic regressions with each data set, and pooled the results for final analyses [34].

To evaluate the mental health impacts of participating in CHAMindWell, we performed a McNemar exact test estimating the change in participants’ odds of having tier 1 (nonclinically relevant) symptoms between baseline and 3-6-month follow-up. We also conducted paired sample *t* tests to evaluate within-subject changes in anxiety, depression, and PTSD symptom severity scores between baseline and follow-up because we felt those symptom domains were the most likely to be improved through participation in CHAMindWell. Because participants in tiers 2 and 3 had more acute needs for symptom reduction and received more interventions than those in tier 1, we repeated these *t* tests for the subsample of participants assigned to tiers 2 and 3 at baseline.

To evaluate whether symptom reductions may have been caused by the natural easing of pandemic-related restrictions and stressors over time, we tested for associations between symptom severity scores and time since the COVID-19 lockdown that surveys were completed among baseline (n=623) and follow-up surveys (n=196). To that end, we conducted 6 regression analyses (3 symptom domains × 2 survey populations) using CAT-MH severity scores for depression, anxiety, and PTSD as the dependent variables and the number of days after COVID-19 lockdown that surveys were completed as the independent variables. We also examined outcomes from a 6-month sample of mental wellness check-ins conducted between September 2020 and March 2021 and calculated the proportions of referrals made to different types of services. All analyses were conducted in RStudio (version 2022.07.2; R Core Team) [35].

### Ethical Considerations

The Cambridge Health Alliance institutional review board office indicated that this program evaluation met the criteria for a quality improvement initiative that did not require informed consent.

## Results

### Overview

**Figure 1:**
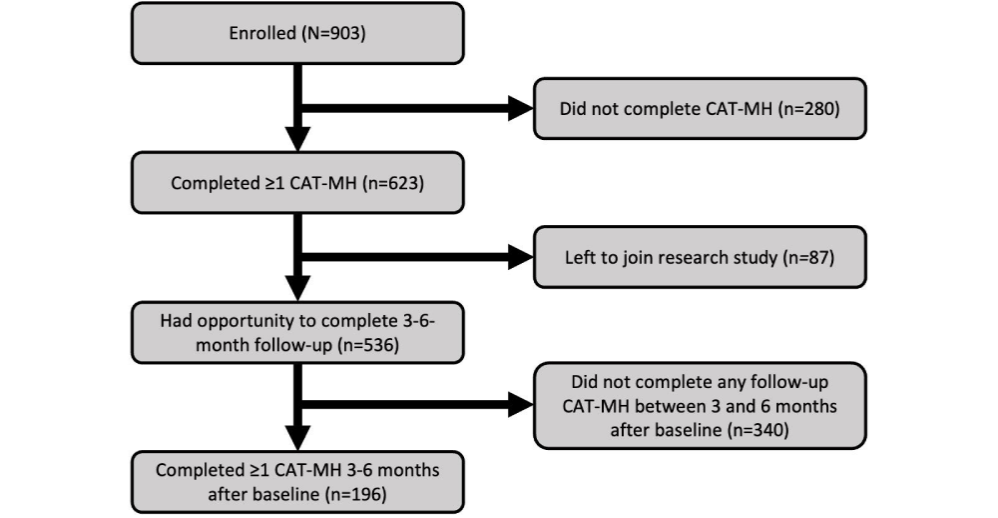
Consolidated Standards of Reporting Trials (CONSORT) diagram for CHAMindWell program evaluation. CAT-MH: Computerized Adaptive Testing for Mental Health.

As Figure 1 illustrates, 623 (69%) of the 903 participants who enrolled in CHAMindWell completed at least 1 CAT-MH assessment, and 87 of those transitioned out of CHAMindWell to join a research study before they had the opportunity to complete a 3-month follow-up survey. Of the 536 who had the opportunity, 196 (36.6%) completed a 3-6-month follow-up CAT-MH survey. Table 1 displays the demographic characteristics of participants by program engagement status.

### Program Engagement

Logistic regressions revealed that White racial identity was the only variable significantly associated with completing a baseline CAT-MH assessment in both complete case analysis (odds ratio [OR] 1.87, 95% CI 1.13-3.10; *P*=.02) and after imputing missing data (OR 1.80, 95% CI 1.14-2.84; *P*=.01). Using complete case analysis, age was the only variable associated with completing follow-up, and the effect size was notably small (OR 1.03, 95% CI 1.01-1.04; *P*=.003). After imputing missing data, no demographic variables were significantly associated with completing follow-up.

### Overall Symptomatology

At baseline, 108 (55.1%) of the 196 participants who went on to complete a 3-6-month follow-up survey had clinically significant symptoms in at least one domain and were assigned to tier 2 or 3. Mean baseline severity scores for each domain were: mean_depression_ 49.4 (SD 17.0), mean_anxiety_ 40.8 (SD 19.3), mean_PTSD_ 41.7 (SD 17.4), mean_mania/hypomania_ 24.6 (SD 20.6), mean_substance use disorder_ 44.0 (SD 15.0), and mean_psychosis_ 30.9 (SD 14.2). Of the 108 in tier 2 or 3 at baseline, 39 (36.1%) no longer had clinically significant symptom severity in any of the domains measured at follow-up. A significant McNemar exact test revealed that participants’ odds of having mental health symptom severity below the clinical threshold were improved by 2.6 times at follow-up (OR 2.60, 95% CI 1.40-5.08; *P*=.001).

### Changes in Anxiety, Depression, and PTSD Symptomatology

As Table 2 illustrates, there were significant reductions in depression, anxiety, and PTSD symptom severity across the entire sample and among those reporting clinically significant symptoms at baseline (ie, tiers 2 and 3). Regression analyses revealed no significant associations between symptom severity and survey completion date among baseline or follow-up surveys for depression (baseline *P*=.31; follow-up *P*=.12), anxiety (baseline *P*=.47; follow-up *P*=.97), or PTSD (baseline *P*=.99; follow-up *P*=.10).

**Table 2:** Changes in Computerized Adaptive Testing for Mental Health (CAT-MH) score between baseline and follow-up among the full sample (n=196) and those in tiers 2 and 3 at baseline (n=108).

CAT-MH scale		Baseline severity score, mean (SD)	Follow-up severity score, mean (SD)	Percent change, %	Effect size, Cohen *d* (95% CI)	*P* value
**Overall sample (n=196)**						
	Depression	49.4 (17.0)	43.7 (20.5)	–11.6	0.30 (0.19-0.41)	<.001
	Anxiety	40.7 (19.4)	35.3 (20.5)	–13.4	0.27 (0.15-0.39)	<.001
	PTSD^a^	41.7 (17.5)	35.9 (19.9)	–13.9	0.31 (0.19-0.43)	<.001
**Baseline tiers 2 and 3 (n=108)**						
	Depression	60.2 (14.3)	52.4 (20.3)	–13	0.42 (0.26-0.59)	<.001
	Anxiety	52.6 (16.1)	48.6 (18.9)	–15.9	0.44 (0.24-0.64)	<.001
	PTSD^a^	51.3 (15.3)	44.6 (19.7)	–13	0.37 (0.18-0.56)	<.001

^a^PTSD: posttraumatic stress disorder.

### Mental Wellness Check-In Outcomes

Between September 2021 and March 2022, a total of 95 individuals in tier 2 or 3 completed a check-in with a CHAMindWell mental wellness clinician. Of those 95 participants, 37 (38.9%) were referred to mindfulness-based group therapy, 9 (9.5%) were referred to other group therapy services, 17 (17.9%) were referred to individual-level services (ie, psychotherapy or psychopharmacology), and 32 (33.7%) did not receive any additional services besides those offered by CHAMindWell (eg, brief coaching, psychoeducation, and guidance on use of self-help apps).

## Discussion

### Overview

CHAMindWell used a stratified care approach to population mental wellness that was associated with reduced severity of mental health symptoms and reduced prevalence of moderate to severe symptom levels among program participants. To our knowledge, this was the first program evaluation to examine the effects of a population mental wellness program designed to promote recovery among those with clinically significant mental health symptoms while preventing the onset of clinically significant symptoms among healthy participants. This program integrated regular mental health screenings with mindfulness-informed mental wellness coaching and treatment referrals. Participants were significantly less likely to report clinically relevant symptom severity after 3-6 months of program engagement and reported significant reductions in symptoms of anxiety, depression, and PTSD at follow-up. We also observed greater symptom reductions among those who reported more severe symptoms at baseline. Furthermore, participants were infrequently referred to individual-level mental health treatments, suggesting that CHAMindWell was associated with improved patient outcomes while minimizing the burden placed on mental health service systems.

While the observed findings could reflect natural symptom reductions caused by the easing of pandemic-related stressors and a return to prepandemic symptomatology, we found no significant associations between dates of survey completion and depression, anxiety, or PTSD symptom severity among baseline or follow-up surveys. These null findings suggest that the observed reductions were not primarily driven by the easing of pandemic-related stressors over time. On the other hand, the lack of linear associations could also reflect multiple rolling periods of peak stress intensity during the 15-month data collection period.

With respect to program engagement, the demographic characteristics of individuals who enrolled in CHAMindWell appear similar to those of the patient population Cambridge Health Alliance (CHA) serves [36]. This suggests that CHAMindWell is, at least in theory, appealing to CHA’s diverse patient population. However, we found that slightly under one-third of enrolled participants (280/903, 31%) did not complete a baseline survey, and White identity was associated with increased odds of baseline survey completion.

There are a variety of factors that may have contributed to this finding. For example, mistrust of health providers may impact the willingness of some in historically underserved communities to self-report mental health symptoms through web-based surveys [37]. Alternatively, some participants may have found the surveys too burdensome or impersonal, and others may have had mental health symptoms that interfered with their ability to complete surveys [38]. In addition, previous research has found that participants who are enrolled digitally may be less likely to complete follow-up than those who are enrolled in person [39]. Finally, a majority of the patients in our hospital system are multilingual or do not speak English, so some may have had difficulty interacting with English-based emails and surveys.

### Limitations

As a program evaluation, this project was not designed to establish causality, for which a more rigorous experimental design, including a control group, would be needed. In addition, the program experienced substantial attrition in the follow-up sample, and the majority of participants identified as White, which may impact the generalizability of our findings to regions with greater racial diversity.

There are also practical limitations associated with implementing a population mental wellness program like CHAMindWell. For example, this program relied on participants self-reporting their mental health symptoms through web-based surveys. As such, individuals with limited access to the internet or internet-connected devices may not have been able to participate. Furthermore, all aspects of this program, including surveys and communications, were conducted in English, which may have limited some participants’ abilities to effectively engage with the program and self-report their symptoms. These factors could impact the generalizability of our findings and the usefulness of this intervention when working with certain populations. To begin addressing these concerns, our team has implemented and is beginning to evaluate a newly developed version of CHAMindWell in Spanish (CHAMindWell en Español).

Finally, this program evaluation used CAT-MH completion as a measure of engagement in CHAMindWell, which does not capture engagement with the recommended resources within each tier. Measuring engagement with and adherence to specific resources and clinician recommendations in the future will help us better understand the applied effects of CHAMindWell.

### Conclusions

CHAMindWell is an integrative population mental wellness program that is associated with reductions in the severity of mental health symptoms among participants while limiting referrals to individual-level services. Such programs may help mental health care systems meet increased treatment needs among their patients without overburdening their providers. Future work should aim to better characterize and address the factors that contribute to program nonengagement and attrition and compare the impacts of CHAMindWell to a control condition.

## Abbreviations

CAT-MH: Computerized Adaptive Testing for Mental Health
CHA: Cambridge Health Alliance
OR: odds ratio
PTSD: posttraumatic stress disorder
REDCap: Research Electronic Data Capture
